# Inflammatory Presentation of a Primary Extranodal Diffuse Large B-cell Lymphoma of the Maxillary Sinus

**DOI:** 10.7759/cureus.38008

**Published:** 2023-04-23

**Authors:** Érica Cerqueira, Margarida Colino, Rui Almeida, Carolina Afonso, Teresa Lopes

**Affiliations:** 1 Maxillofacial Surgery, Centro Hospitalar Universitário de Coimbra, Coimbra, PRT; 2 Pathology, Centro Hospitalar Universitário de Coimbra, Coimbra, PRT; 3 Hematology, Centro Hospitalar Universitário de Coimbra, Coimbra, PRT

**Keywords:** dlbcl, non-hodgkin lymphoma, extra nodal, oncology, maxillary sinus, diffuse large b-cell lymphoma

## Abstract

Diffuse large B-cell lymphoma originating from the maxillary sinus is rarely reported in the literature. Diagnosis is challenging since the long absence of signs and symptoms allows it to grow undetected or be confused with benign inflammatory conditions. The purpose of this paper is to present an unusual manifestation of this rare pathology. A patient in his 50s, presented to his local emergency department with malar and left eye pain after local trauma. Physical examination showed infraorbital edema, palpebral ptosis, exophthalmos, and left ophthalmoplegia. CT scan showed a soft tissue mass measuring 43x31 mm in the left maxillary sinus. An incisional biopsy was performed, and results showed diffuse large B-cell lymphoma with positivity for CD10^+^, BCL6^+^, BCL2^+^, and Ki-67 index greater than 95%. Afterward, the patient promptly started treatment with rituximab-cyclophosphamide-hydroxydaunorubicin-Oncovin-prednisone (R-CHOP) chemotherapy. A good medical history, clinical and imaging evaluations, and anatomopathological studies are crucial to establish an early diagnosis of diffuse large B-cell lymphoma (DLBCL).

## Introduction

Diffuse large B-cell lymphoma (DLBCL) is the most common type of non-Hodgkin lymphoma (NHL), and its primary involvement of the maxillary sinus has rarely been reported in the literature. This type of presentation can often pose a diagnostic challenge [1-3]. DLBCL often involves extranodal sites within the head and neck region, with the most common involvement being the Waldeyer’s ring [3-6]. Additionally, nasal cavity, paranasal sinuses, orbital cavity, thyroid gland, and salivary glands can also be affected [3,5]. Regarding primary paranasal sinus DLBCL, the prolonged absence of signs and symptoms allows it to grow undetected for an extended period or to be confused with benign inflammatory conditions and upper respiratory infections, therefore delaying a correct diagnosis [1,5]. Nasal obstruction, rhinorrhea, facial swelling, or visual symptoms are examples of possible clinical presentations [3,5]. Early diagnosis is challenging, and due to its specific anatomy, patients can be asymptomatic until the tumor infiltrates one of the maxillary sinus walls. A delayed diagnosis increase significantly morbidity and mortality in these patients [1,4]. This study aimed to present an unusual manifestation of a rare pathology, namely primary DLBCL of the maxillary sinus.

## Case presentation

**Figure 1:**
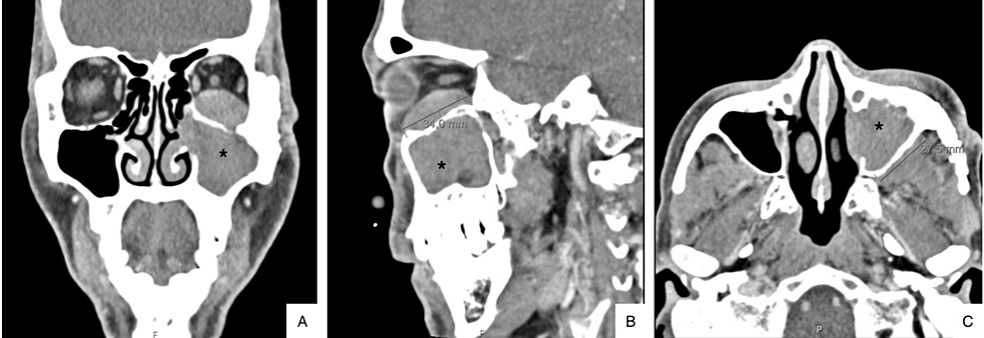
CT scan coronal, sagittal, and axial views showed an extensive soft tissue mass in the left maxillary sinus which extended through the left orbit (asterisk). (A) coronal view, (B) sagittal view, and (C) axial view

A patient in his 50s initially visited his local emergency department, complaining of pain in the left eye and cheek in the last week after sustaining a local trauma with a tree branch. His medical history revealed a previous open reduction and osteosynthesis of a left zygomatic arch fracture about 20 years ago. On examination, he presented with an ulcerated lesion at the same site as the previous surgical scar, accompanied by slight redness and left infraorbital edema. A maxillofacial CT scan was performed, which showed well-placed and stable osteosynthesis material without any new fractures. Additionally, a soft tissue mass measuring approximately 43x31 mm in size was identified, centered in the left maxillary sinus (Figures 1A-1C).

Due to the suspicion of an inflammatory cause related to the recent trauma over a previous fracture osteosynthesis, the patient was prescribed oral antibiotics and anti-inflammatory drugs. However, a week later, he was referred to our outpatient department for re-evaluation as he reported no improvement and complained of persistent symptoms. On physical examination, an ulcerated malar lesion was observed along with associated infraorbital edema, palpebral ptosis, exophthalmos, and left ophthalmoplegia.

Based on the patient's clinical presentation and previous CT scan, the differential diagnosis included acute infection of the maxillary sinus, delayed inflammatory reaction to osteosynthesis material, or a neoplastic lesion. As the patient did not show any improvement with oral antibiotics, he was admitted to our inpatient department for intravenous antibiotic and corticosteroid therapy and further investigation. Although there was a slight improvement in the swelling in the first few days, ophthalmoplegia persisted almost unchanged. Therefore, the medical team decided to perform an incisional biopsy of the lesion in the maxillary sinus for further diagnosis.

**Figure 2:**
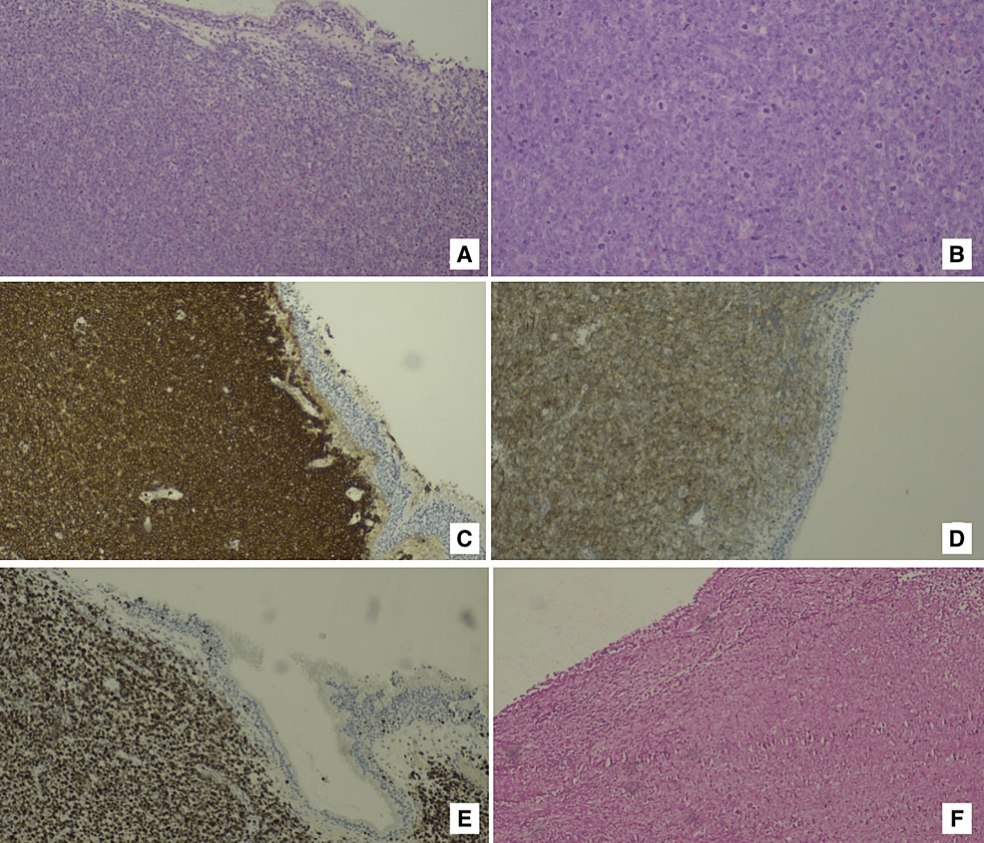
Histopathological examination of tissue sections. (A) H&E 100x: diffuse pattern lymphoproliferative neoplasm presenting under preserved maxillary sinus mucosa; (B) H&E 200x: neoplasm composed by large lymphocytes, with mitosis and apoptosis images; (C) CD20 100x: neoplastic cells with immunohistochemical positivity, a B lymphocyte marker; (D) CD10 100x: neoplastic cells with immunohistochemical expression, a germinal center marker; (E) Ki67 100x: neoplastic cells with high proliferative index; (F) EBER 100x: neoplastic cells without evidence of Epstein-Barr virus infection, evaluated by in situ hybridization. H&E: hematoxylin and eosin; EBER: Epstein-Barr encoding region

The histopathological examination of tissue sections revealed a diffuse pattern neoplasm with large lymphocytes, numerous mitoses, and apoptosis (Figures 2A-2F). Immunohistochemical studies demonstrated expression of CD20, CD10, BCL6, BCL2, MUM1, and c-MYC in the neoplastic cells. The proliferative index, as evaluated by Ki-67, was greater than 95%, while Epstein-Barr virus (EBV)-encoded small RNA (EBER) in situ hybridization was negative. These findings supported the diagnosis of DLBCL, germinal center B-cell-like subtype (GCB). Blood serologies were negative for hepatitis B virus (HBV), hepatitis C virus (HCV), human immunodeficiency virus (HIV), EBV, and Cytomegalovirus (CMV) and showed immunity to herpes simplex 1/2. The patient underwent staging with a bone marrow biopsy and a positron emission tomography-computed tomography (PET-CT) scan, which showed no signs of lymphoma involvement in the bone marrow. The PET-CT scan confirmed the presence of a highly hypermetabolic soft tissue mass measuring approximately 43x31 mm, centered on the left maxillary sinus, with supradiaphragmatic lymphomatous lymph node involvement.

After the diagnosis, the patient was referred to the hematology department, where he started treatment with rituximab-cyclophosphamide-hydroxydaunorubicin-Oncovin-prednisone (R-CHOP) chemotherapy. The proposed treatment for this patient was R-CHOP, for a maximum of six cycles, followed by high-dose methotrexate during hospitalization to prevent dissemination to the CNS. Unfortunately, the patient abandoned the treatment.

## Discussion

DLBCL is the most common subtype of non-Hodgkin lymphoma, which can involve nodal and extranodal organs and tissues [7]. Extranodal presentation represents about 20-30% of NHLs [8,9]. Extranodal NHLs originating from the oral and perioral regions are uncommon, and maxillary sinus presentation is extremely rare and often asymptomatic [1-3]. When symptomatic, it frequently simulates inflammatory disease. Non-specific symptoms make the diagnosis of these neoplasms extremely challenging.

In the reported case, the patient initially complained of acute malar painful swelling, which he disregarded and assumed was related to the recent trauma. Noticeable palpebral ptosis and left ophthalmoplegia were possibly compatible with a traumatic cause even though no fractures were clinically identifiable. The absence of systemic symptoms (such as fever, weight loss, or night sweats) and the presence of acute inflammatory signs led us to consider an acute maxillary infection as the first diagnostic hypothesis. Therefore, anti-inflammatory and antibiotic therapy was instituted. After performing the maxillofacial CT scan, a mass that occupied the entire sinus was identified without expansion or destruction of the walls. After only slight improvement with intravenous therapy, other diagnostic hypotheses of non-inflammatory causes were raised, and a biopsy was performed three weeks after the initial presentation.

Histopathological and immunohistochemical examination rendered the definitive diagnosis of DLBCL [9,10]. The expression of CD10, BCL6, and MUM1 allows classification between GCB lymphoma and non-GCB lymphoma [6]. The immunohistochemical expression of CD10, as shown in this case, enables classification as a GCB lymphoma, which implies a relatively better prognosis [2]. DLBCL is an aggressive tumor and is rapidly fatal without treatment. Therefore, early diagnosis plays an important role in the prognosis of these patients. Rituximab, an anti-CD20 medication, has been found to increase the likelihood of complete remission in patients with non-Hodgkin's lymphoma. Additionally, it appears to extend the overall survival of patients. However, it should be noted that around 35% of patients with DLBCL lymphoma who have CD20^+^ cells do not respond to R-CHOP therapy [2,7].

Additional studies with cytogenetics should have been performed to avoid missing a case of high-grade B-cell lymphoma with MYC and BCL2 rearrangements. This is a more aggressive type of B-cell lymphoma characterized by these gene rearrangements, normally with a germinal center immunoprofile and high proliferative index. They have a worse response to R-CHOP compared to DLBCL [11,12]. These cytogenetic studies were not performed because of the loss of follow-up.

There are other similar cases reported in the literature, although the majority are non-GCB subtypes. Almost all of the patients achieved a complete response at the end of chemotherapy, some receiving CHOP and others R-CHOP. The majority of patients remained in total remission during the follow-up period [2,13-15]. This data allows us to suspect that the patient in this study would have a good chance of complete remission after undergoing cycles of R-CHOP, even more, if we consider that GCB subtype DLBCL has a better response to chemotherapy and an overall better outcome, compared to the non-GCB.

## Conclusions

In conclusion, the importance of this study relies on the description of an initial trauma patient with an added confounding factor of previous trauma to the same site and previous surgery with open reduction and osteosynthesis. This situation should remind physicians that a neoplastic lesion may mimic a typically inflammatory presentation, even when the history is consistent. It is therefore important to raise special awareness if the patient does not improve with antibiotics and anti-inflammatory therapy, and other etiologies should be excluded. Close attention to patient manifestations and evolution, as well as imagiological and histological findings, is encouraged to achieve an accurate and rapid diagnosis, precise treatments, and better prognosis.
